# Subcutaneous Quadrantectomy Is a Safe Procedure in Management of Early-Stage Breast Cancer

**DOI:** 10.3389/fsurg.2022.829975

**Published:** 2022-04-15

**Authors:** Eva Lieto, Annamaria Auricchio, Silvia Erario, Giovanni Del Sorbo, Francesca Cardella

**Affiliations:** Oncologic Surgery Unit, Translational Medicine Department, University of Campania Luigi Vanvitelli, Naples, Italy

**Keywords:** breast surgery, subcutaneous quadrantectomy, mini-invasive breast surgery, breast cancer treatment, breast cancer surgical technique

## Abstract

**Background:**

A less-invasive surgery is often required today for many tumors, when oncologic radicality is strictly ensured, both to minimize hospital stay and health costs and to guarantee aesthetical results. Breast surgery for cancer has been radically changed in the last years since conservative interventions are widely performed everywhere.

**Methods:**

The authors present 75 cases of early breast cancer, randomly treated with standard quadrantectomy and subcutaneous quadrantectomy; the totally subcutaneous surgical technique implies only a short periareolar skin incision and a complete quadrant resection with skin and subcutaneous layer preservation. Continuous data were analyzed by unpaired Student's *t*-test. The Chi-square test was used to cumulate categorical variables. The Kaplan–Meyer method and log-rank test were used to compare the overall survival and disease-free survival.

**Results:**

No difference was found among the two groups in terms of the type of tumor, overall survival (OS), disease-free survival (DFS), early complications, radicality, and mortality. The only significant differences were both found in the length of hospital stay and in postoperative breast deformity that required further intervention in some cases.

**Conclusion:**

In the era of mini-invasive surgery and quality assurance, the authors conclude that subcutaneous quadrantectomy is a safe procedure that allows less health cost and a better aesthetical result.

## Introduction

Breast cancer is the most frequent cancer in women, with a whole estimated incidence of 255,000 new cases per year in the USA (1); the mortality rate is declining in the last two decades with a specific survival rate ranging from 74 to 82%.

These encouraging results are due either to effective population screening programs or to the huge progress in cancer therapy and management (2), especially among young women.

Since its high incidence, breast cancer is a real public health issue, consuming about 13% of the total cancer healthcare cost (3). This is the reason why many efforts are made nowadays to improve clinical results, with the same effectiveness, also in terms of reduction of hospitalization and request of further surgical appeals.

The concept of radicality in breast cancer has been deeply modified in the last years, going through widely demolition interventions toward a breast conservative surgery (4, 5); also, the axillary approach has changed at the same time, being axillary dissection a need and not a choice, today (6).

In the era of mini-invasive surgery, safety and an aesthetic outcome can walk together to ensure the patient a good oncologic result and a good quality of life.

Due to the diffusion of screening programs, a large number of early breast cancers are diagnosed today; consequently, a less-invasive radical surgery is mandatory. Subcutaneous endoscopic mastectomy (7) is one of the current challenges in that type of surgery, even if it takes more time to finally standardize the technique.

On the other hand, subcutaneous breast demolition surgery, in which the skin covering is preserved to obtain better plastic reconstruction, can be considered the standard of care in the surgical management of breast cancer today (8, 9); the so-called oncoplasty (10) is the multimodal treatment that implies contemporary demolition and reconstructive surgery in the aim to ensure the women a good quality of life, even after an intervention that can be perceived as mutilation (11–14). The technique of subcutaneous resection is well-known and is widely applied in breast surgery; unfortunately, too many standard breast resections, with correspondent skin removal, are still performed today, because they are judged to be more complete in terms of oncologic radicality, for the full-thickness tissue removal, and the easier control of the width of the resection about the tumor position.

We retain that when small breast cancer is finally encompassed into the mammalian gland, its radical removal is possible by a glandular quadrantectomy, along an avascular plane with a subcutaneous layer, through a small periareolar skin incision. We propose the results of totally subcutaneous quadrantectomy (TSQ) in comparison with standard quadrantectomy (SQ) in early-stage breast cancer in terms of OS and DFS. Subcutaneous quadrantectomy is not introduced as an oncoplastic technique; this operation, where no plastic reconstruction is required, could be considered as a kind of mini-invasive breast resection in which aesthetical advantage is achieved without compromising the oncological radicality.

## Materials and Methods

### Patients

From January 1, 2016, to December 31, 2020, 75 patients with early-stage (T1/T2-N0) breast cancer were surgically treated in a single-center unit of the Vanvitelli University of Campania.

Inclusion criteria were represented by histologically proven breast cancer, tumor size ranging between <1 cm and 3 cm, non-metastatic sentinel node status, peripheral (non-retroareolar) position of the tumor into the breast, and absence of surrounding tissues' tumoral spreading signs.

All the patients were staged as follows: anamnesis and clinical examination, mammography, breast/axillary ultrasonography, Fine Needle Aspiration Biopsy (FNAB)/core biopsy of the tumor, chest radiography, liver ultrasonography, whole-body scintigraphy, to confirm early-stage breast cancer; more sensitive imaging techniques, such as chest/abdomen CT scan and/or MRI, were used in doubtful cases.

All the patients gave their informed consent, and they were aware to undergo one of the two surgical procedures. The study was approved by the Ethical Committee of Vanvitelli University.

### Surgical Procedure

All operations were performed under general anesthesia. All the patients initially underwent sentinel node biopsy (SNB); as, usually, a ^99^Tc-labeled human serum albumin lymphoscintigraphy is carried out 3 h before surgery, and the position of the sentinel node is signed over the skin; harvesting of sentinel node/s is performed with the aim of an intraoperative handheld gamma-detection probe, and a sample is extemporary examined; cases of metastatic sentinel nodes, in which axillary dissection was required, were excluded from this study.

Randomization was performed by opening a closed envelope in an operating theater, unequivocally assigning the patient to undergo SQ or TSQ.

The SQ was done through an elliptical skin incision in correspondence of the interested quadrant and subsequently removing an entire segment, including skin, subcutaneous layer, mammalian gland, and corresponding pectoral fascia.

The TSQ was conversely performed through a 2-cm periareolar incision at the tumor position into the breast. A completely avascular plane is to be found between a gland capsule and a subcutaneous layer so that this one is spared for a better aesthetical result.

The technique of quadrantectomy, both in standard and in subcutaneous resection, consists of the complete removal of the quadrant in which the tumor is decompressed; while, in SQ, the specimen includes the correspondent skin surface, in TSQ, through a 2-cm periareolar skin incision, the same elliptic area of the breast is gradually detached from the subcutaneous coat, resected from the remnant gland, and, finally, removed from the muscular layer. After quadrantectomy, the edges of the remnant gland are precisely juxtaposed by several stitches; finally, the subcutaneous layer and skin are aesthetically sutured.

Drainages were never used in both groups, and only a compressive bandage was left in place at the end of the operation; all the patients were advised to use a compressive bra for almost 2 weeks.

### Statistical Analysis

Statistical analyses were carried out using the SPSS 20.0 software (SPSS Inc., Chicago, IL, USA).

Continuous data are expressed as a range and mean ± SD values. The equality of the group means was analyzed by unpaired Student's *t*-test. The Chi-square test was used to analyze correlations between categorical variables. The Kaplan–Meier method and long-rank test were used to compare survival curves. The OS rate was calculated from the date of surgery to the end of follow-up or death for any cause. On the contrary, the DFS rate was calculated from the date of surgery to the date of tumor relapse by censoring the patients who died for causes other than breast cancer. All analyses were two-sided; *p* < 0.05 was considered to be statistically significant.

These formatting styles are meant as a guide, as long as the heading levels are clear; Frontier's style will be applied during typesetting.

## Result

According to the randomization, 38 patients were assigned to SQ (Group A), with full-thickness tissue removal, and 37 patients underwent TSQ (Group B), in which resection was only limited to the gland, respectively. All the operations were successful without the need to change the programmed technique.

The two groups matched well; particularly, they did not show any difference in size, node metastasis, radicality, postoperative complications, hormone receptor, and human epidermal growth factor receptor 2 (HER-2) status (Table 1). On the contrary, the patients undergoing SQ experienced a more significant number of breast deformities than the patients undergoing TSQ (31 vs. 3%).

**Table 1:** Characteristics of the series and results.

	**SQ (Group A)**	**TSQ (Group B)**	***p***
Number	38	37	
Age	34–76	27–77	*0.592^a^*
(years)	54 ± 11	53 ± 12	
Size	0.2–2.8	0.5–2.7	*0.423^a^*
(cm)	1.4 ± 0.7	1.6 ± 0.6	
Node positive	2 (5)	2 (5)	*0.978^b^*
Radicality (%)	100	100	*NA*
Hospital stay	3–10	2–7	*0.001*
(days)	6 ± 2	4 ± 1	
Postoperative complications	5 (13)	3 (8)	*0.479^b^*
Wound infection	2 (5)	2 (5)	
Seroma	3 (8)	1 (3)	
**Hormone receptor status**
Positive	31 (82)	32 (87)	*0.562^b^*
Negative	7 (18)	5 (13)	
**Her-2 status**
Positive	9 (23)	9 (25)	*0.94^b^*
Negative	29 (77)	28 (75)	
Breast Deformity	12 (31)	1 (3)	*0.001^b^*
5y Actuarial OS (%)	98%	97%	*HR = 0.91 0.926*
5y Actuarial DFS (%)	92%	95%	*HR = 0.86 0.889*
Cancer mortality	1 (3)	1 (3)	*0.985^b^*

*SQ indicates standard quadrantectomy. TSQ indicates totally subcutaneous quadrantectomy.*

*Age, size, and hospital stay are expressed as a range, and mean ± standard deviation values.*

*Numbers in parentheses are percentages.*

*The Kaplan–Meier method and long-rank test were used to compare overall survival (OS) and disease-free survival (DFS) rates.*

*HR indicates hazard ratio.*

a *Student's t-test.*

b *Chi-square test*.

All non-surgical complications, namely wound infection and serum collection, were treated conservatively with antibiotics and multiple collection drainages. Interestingly, in Group A, 5 out of 12 scar retractions with breast deformity required further corrective operation. Since the absence of remnant “empty spaces,” plastic surgery is hardly ever required; in normal- to large-size breasts, TSQ is generally enough to obtain a good aesthetical result, with scarce or minimal breast asymmetry; in small breasts, conversely, a filling intervention has been delayed, if necessary, at the scar stabilization, few months after the operation.

After the surgery, all the patients underwent local radiotherapy and were given hormone therapy when required.

**Figure 1:**
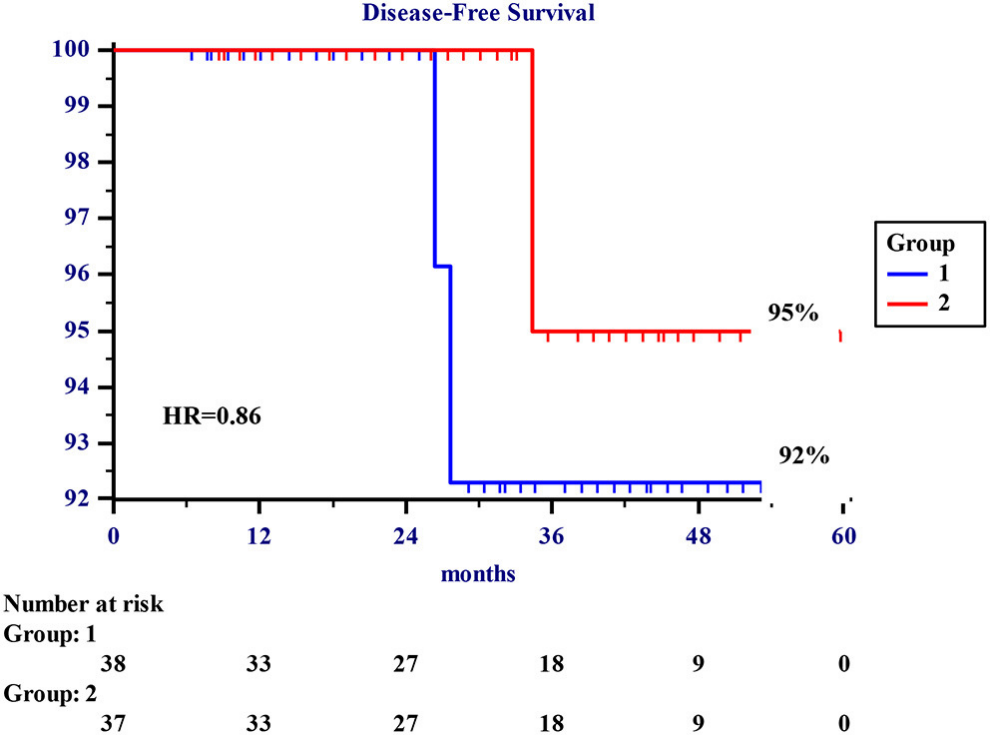
About 5-year actuarial disease-free survival (DFS). About 1–5-year disease-free survival rates in 75 patients undergoing surgery for early breast cancer. Group 1 refers to 38 patients undergoing standard quadrantectomy. Group 2 refers to 37 patients undergoing totally subcutaneous quadrantectomy.

No patient was lost to the follow-up, and it was completed on June 30, 2021. There was no difference in the oncological outcome in both groups. The 5-year actuarial overall survival rates were 98 and 97%, Groups A and B, respectively (Figure 1). The 5-year DFS rates were 92 and 95%, respectively. One patient in each group (2.6 and 2.7%, respectively) died of breast cancer.

Hospital stay and breast deformity were significantly different in the two groups, with *p* < 0.001.

## Discussion

A less-invasive surgery is one of the principal aims of the current practice to issue the same result in terms of radicality, with minimum discomfort for the patient.

The recent history of breast cancer surgery has followed this line since it is well-known today that breast conservative surgery is completely stackable to more demolition surgical interventions in terms of oncologic radicality and life expectation (8).

The effectiveness and safety of subcutaneous surgery, limited to the mammalian gland, arises from the experience in skin-sparing and nipple-sparing mastectomy, with immediate prosthetic replacement, both in prophylactic and curative settings. The oncoplastic surgery, in which plastic surgical techniques of replacement or reconstruction of the breast are done during the demolition operation, is the standard of care in breast cancer management (10); the technique we are proposing herein is a sort of mini-invasive quadrantectomy, with better aesthetical results and no difference in terms of oncological radicality. When the tumor is completely contained in the capsule, a TSQ is as curative as an SQ with surely better functional and aesthetical results. In early-stage breast cancers, often arising in young women, we retain that a mini-invasive surgery should be given without compromising any long-term result.

Since the skin is preserved and the underlying remnant gland is immediately reconstructed by the juxtaposition of the edges, no residual “space” may induce deformities in the breast shape, thus, no further oncoplastic reconstruction is required. For small breast cancers, a radical resection can be achieved also through limited resections with a free margin of 1–2 mm (8). In the subcutaneous quadrantectomy, the skin is preserved, while the width of the resection is as similar as that of the standard one; in such selected cases, removing the skin surface in the corresponding area of the tumor is not useful, in terms of oncological radicality, and detrimental, in terms of aesthetical results. The correct subcutaneous resection, with complete preservation of the skin and the fatty layer between dermis and gland capsule, is the true challenge of this technique, instead. The subcutaneous approach may be so arduous that many surgeons are often induced to avoid it, just because of the risk of necrotizing lesions of the skin, due to an imperfect subcutaneous dissection (15); this fact is particularly true when the tumor is far from the areola and the skin incision is short. Furthermore, the subcutaneous resection surely requires a greater experience by the surgeon than the standard one, because the accuracy and radicality of the resection must be ensured through a narrow skin incision, and, during the operation, the surgeon must continuously keep in mind the tridimensional vision of the specimen he is removing. Moreover, there are rare risk conditions (i.e., re-intervention, previous breast inflammatory diseases) where subcutaneous dissection may be particularly difficult (16), in which the surgeon can choose the best technique from time to time.

**Figure 2:**
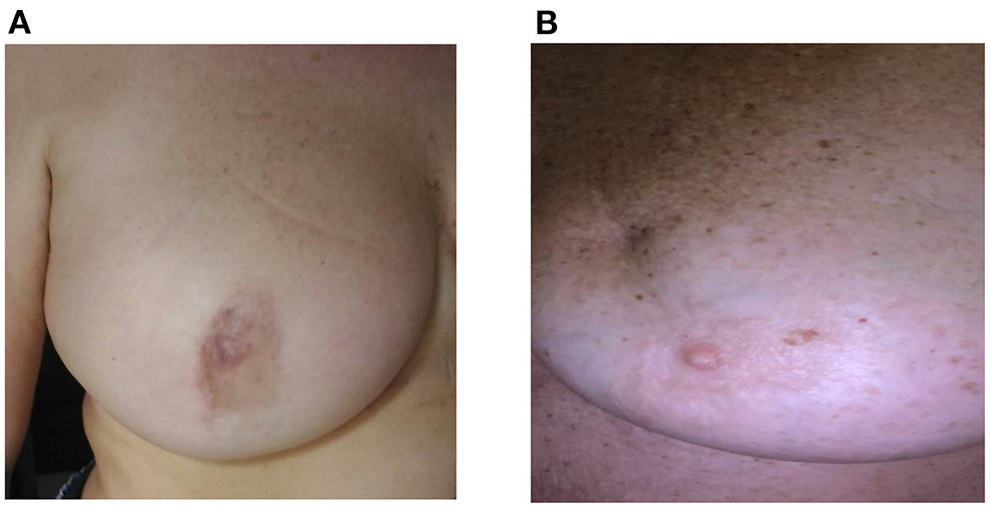
**(A,B)** Upper-external quadrantectomy: long-term results. **(A)** totally subcutaneous quadrantectomy (TSQ): The scar is near the areola with minimal alteration of the breast shape. **(B)** standard quadrantectomy (SQ): The scar is into the quadrant with minimal retraction in the middle third.

No difference was found in terms of incidence of early complications (i.e., wound infection and seroma) between TSQ and SQ; conversely, the breast deformity due to the scar retraction is significantly more frequent in SQ than in TSQ (Table 1); since the integrity of the integuments at the site of glandular resection, the TSQ is less frequently complicated with a retracting scar; only one case of breast deformity due to glandular unstitching in an early postoperative period after wound infection was registered in our series. In most cases, imperceptible changes were observed in a breast profile due to reduction of the gland volume rather than scarring deformities, instead (Figure 2).

In the small series, we have analyzed that OS and DFS are quite the same, with non-significant variations in both groups. Early complications were not significantly different between the two groups. On the contrary, late complications, such as scar retracting often requiring re-operation, were lower in the TSQ group and then in the SQ group, with a better aesthetical result; moreover, any re-operation has been performed in the TSQ group with sensible money-saving in terms of hospitalization and resumption of the patient's normal activities.

The most important limitation of the present study is the small number of the sample due to the exclusive selection of early-stage breast disease, and the single-center experience. A multicentric study should be desirable, in the future, to give the results a strong evidence power.

In conclusion, despite the smallness of the sample, subcutaneous quadrantectomy is a safe procedure in breast cancer management and is strongly recommended in young women in which aesthetical result is mandatory to ensure them a good quality of life.

## Data Availability Statement

The raw data supporting the conclusions of this article will be made available by the authors, without undue reservation.

## Ethics Statement

The studies involving human participants were reviewed and approved by Ethics Committee of Vanvitelli University. The patients/participants provided their written informed consent to participate in this study.

## Author Contributions

EL and AA: design and revision. FC: data interpretation. SE and GS: data acquisition. All authors contributed to the article and approved the submitted version.

## Conflict of Interest

The authors declare that the research was conducted in the absence of any commercial or financial relationships that could be construed as a potential conflict of interest.

## Publisher's Note

All claims expressed in this article are solely those of the authors and do not necessarily represent those of their affiliated organizations, or those of the publisher, the editors and the reviewers. Any product that may be evaluated in this article, or claim that may be made by its manufacturer, is not guaranteed or endorsed by the publisher.
